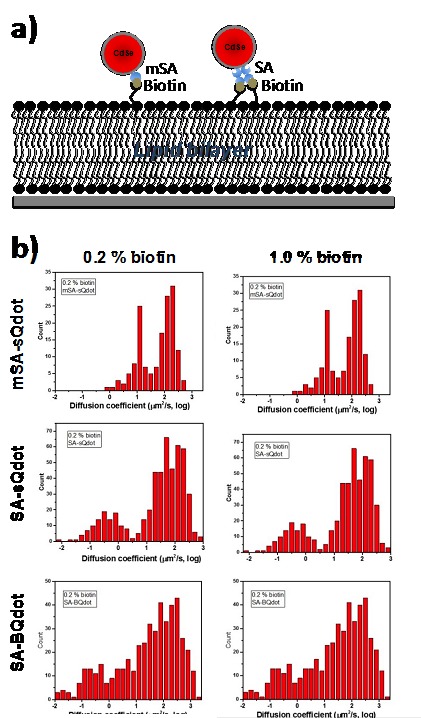# Correction: Super-resolution imaging of synaptic and Extra-synaptic AMPA receptors with different-sized fluorescent probes

**DOI:** 10.7554/eLife.33413

**Published:** 2017-11-08

**Authors:** Sang Hak Lee, Chaoyi Jin, En Cai, Pinghua Ge, Yuji Ishitsuka, Kai Wen Teng, Andre A de Thomaz, Duncan Nall, Murat Baday, Okunola Jeyifous, Daniel Demonte, Christopher M Dundas, Sheldon Park, Jary Y Delgado, William N Green, Paul R Selvin

Lee SH, Jin C, Cai E, Ge P, Ishitsuka Y, Teng KW, de Thomaz AA, Nall D, Baday M, Jeyifous O, Demonte D, Dundas CM, Park S, Delgado JY, Green WN, Selvin PR. 2017. Super-resolution imaging of synaptic and Extra-synaptic AMPA receptors with different-sized fluorescent probes. *eLife* **6**:e27744. doi: 10.7554/eLife.27744.

Published 27, July 2017

There was an error in the histogram of Figure 4–figure supplement 3b, which showed identical histograms at 0.2% and 1.0% biotin conditions. We accidently put the histogram of 0.2% biotin in place of the 1.0% biotin condition. We have now corrected this. Correcting this error does not affect any of the quantitative data reported in the text or the conclusions of the study.

The article has been corrected accordingly.

The corrected Figure 4–figure supplement 3 is shown here:

**Figure 1:**
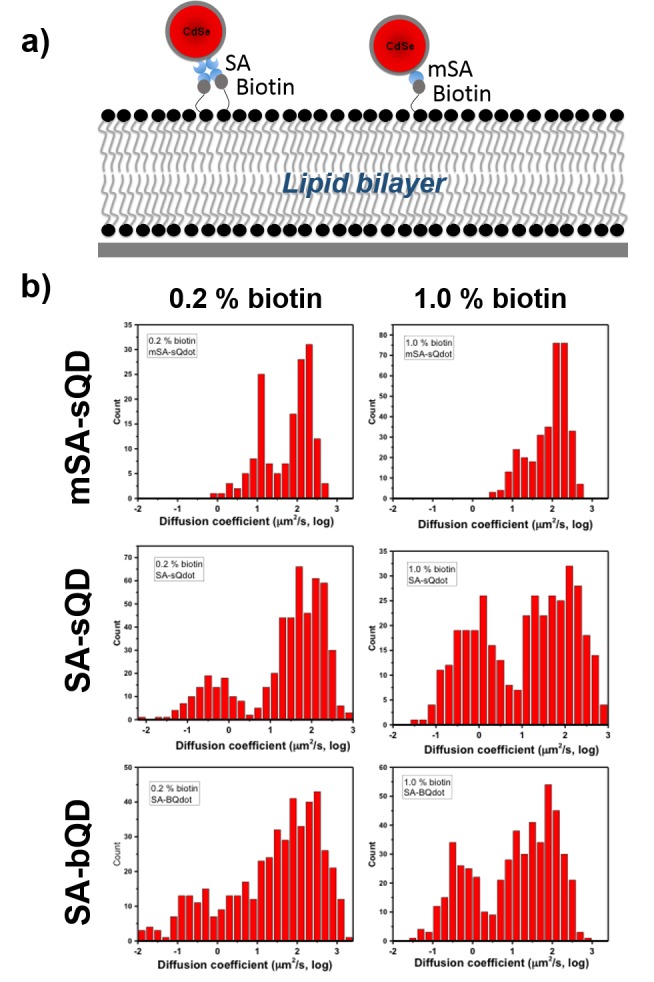


The originally published Figure 4– figure supplement 3 is shown here:

**Figure 2:**